# T84. DO SIMILAR COGNITIVE MECHANISMS ENCOURAGE DELUSION-LIKE IDEATION AND BELIEF IN FAKE NEWS?

**DOI:** 10.1093/schbul/sby016.360

**Published:** 2018-04-01

**Authors:** Michael Bronstein, Gordon Pennycook, Adam Bear, Tyrone Cannon, David Rand

**Affiliations:** Yale University

## Abstract

**Background:**

Increasingly, the positive symptoms of psychosis are recognized as being on a continuum with phenomena that are experienced by many members of the general population (i.e., non-clinical samples). Delusions are no exception. These fixed false beliefs, which are common in individuals with psychosis, are echoed by inflexible false beliefs in the general population that have delusion-like qualities (e.g., belief in clairvoyance). In a series of studies, we sought to determine whether belief in a particular type of disinformation (fake news) might represent a point on the same continuum as delusions and delusion-like ideation. To this end, we examined whether individuals who endorsed more delusion-like ideation were also more prone to believing fake news. We then examined whether the cognitive mechanisms behind any relationship between delusion-like ideation and fake news were similar to those associated with delusion-like ideation generally.

**Methods:**

503 participants were recruited using Amazon’s Mechanical Turk (MTurk). Participants completed a test of ability to discriminate real from fake news along with several individual difference measures. These included measures of delusion-like ideation (the Peters et al. Delusion Inventory [PDI]), engagement in analytic thinking (the Cognitive Reflection Test [CRT]), and the degree to which one values evidence in forming and revising beliefs (the Actively Open-Minded Thinking Questionnaire [AOT]). Mediation tests were conducted using the PROCESS macro for SPSS (model 4, with 5000 bootstrapped samples and bias-corrected 95% confidence intervals).

**Results:**

Delusion-like ideation was positively correlated with belief in fake news (rho(501) = .20, p < .001). The relationship between belief in fake news and delusion-like ideation was partially explained by lower levels of analytic thinking ability (as measured by the CRT; completely standardized 95% CI = [.02 .07]) and lower evidence valuation (as measured by AOT scores; completely standardized 95% CI = [.01 .06]). These indirect effects accounted for 39% of the relationship between delusion-like ideation and belief in fake news. Delusion-like ideation and belief in real news were not correlated (rho(501) = 0.01, p = .927).

**Discussion:**

Consistent with the notion that belief in fake news represents a point on the same continuum as belief in delusional and delusion-like ideas, belief in fake news was associated with increased endorsement of delusion-like ideation. This relationship was partially explained by factors previously associated with delusions and delusion-like ideation (e.g., lower engagement in analytic thinking, lower valuation of evidence in belief formation and revision). The link between delusion-proneness and belief in fake news (which was established for the first time in these studies) may prove useful in helping to inoculate the public against the deleterious effects of purposely-spread misinformation. Identifying individuals who might be at high risk of falling for fake news is an essential first step in this direction. The present results suggest that individuals who endorse delusion-like ideation may be one population toward which interventions aimed at preventing belief in misinformation might usefully be aimed.

